# Effects of Diets Enriched in Linseed and Fish Oil on the Expression Pattern of Toll-Like Receptors 4 and Proinflammatory Cytokines on Gonadal Axis and Reproductive Organs in Rabbit Buck

**DOI:** 10.1155/2020/4327470

**Published:** 2020-01-21

**Authors:** Laura Menchetti, Olimpia Barbato, Monica Sforna, Daniele Vigo, Simona Mattioli, Giulio Curone, Marco Tecilla, Federica Riva, Gabriele Brecchia

**Affiliations:** ^1^Department of Veterinary Medicine, University of Perugia, 06123 Perugia, Italy; ^2^Department of Veterinary Medicine, University of Milano, 20133 Milano, Italy; ^3^Department of Agricultural, Environmental and Food Science, University of Perugia, 06121 Perugia, Italy

## Abstract

Infections of the genital tract can perturb the fertility in humans and animals. Pathogen recognition and activation of innate immunity onset through the pattern recognition receptor activation, such as Toll-like receptor 4 (TLR4), leading to the production of proinflammatory cytokines and mediators. TLR4 is expressed both on leukocytes and nonimmune cells. Rabbit TLR4 shows great similarity to its human counterpart. Moreover, the TLR4 signalling pathway could be modulated by long-chain polyunsaturated fatty acids (LC-PUFA). The objectives of this study were (i) to determine the expression levels of TLR4 and proinflammatory cytokines in the reproductive hypothalamic-gonadal axis of the male rabbit and (ii) to evaluate if the n-3 PUFA-enriched diets can modify their expression levels in the tissues and LC-PUFA profiles in seminal plasma. Fifteen rabbit bucks (*n* = 5/experimental group) were fed with different diets: commercial standard (group C), rich in extruded linseed (10%, group L), and in fish oil (3%, group FO) for 110 days. TLR4, TNF-*α*, and IL-1*β* mRNA were ubiquitously expressed throughout the hypothalamic-gonadal axis. However, TLR4 mRNA expression was lower in the hypothalamus than the epididymis (*P* < 0.01), seminal vesicles (*P* < 0.01), and pituitary gland (*P* < 0.05). Dietary enrichment in PUFA did not modify the gene expression profile nor the histological characteristics of the tissues. Conversely in seminal plasma, rabbits fed with L and FO had lower n-6 (*P* < 0.05), LC-PUFA n-6 (*P* < 0.05), and n-6/n-3 ratio (*P* < 0.05) but higher n-3 (*P* < 0.001) and LC-PUFA n-3 (*P* < 0.01) compared to the control group. Our study builds a map of the gene expression of TRL4 and proinflammatory cytokines in the reproductive hypothalamic-gonadal axis of the male rabbit, fundamental step for understanding the immune defence mechanisms. Diets enriched in LC-PUFA did not affect basal gene expression but modulated sperm fatty acid composition. Finally, rabbit may be an excellent animal model to study the relationship between inflammation and infertility, and the nutritional modulation of immune functions.

## 1. Introduction

Male infertility is increasing in the last decades in both human and animals [1–3]. Infertility can be due to genetic and nongenetic factors. Nongenetic factors consist of andrological problems such as varicocele and cryptorchidism as well as immunological diseases including antisperm antibodies in serum and ejaculation [1]. Nongenetic factors also include the nutrition that can negatively affect the fertility impairing the functions of the gonadal axis both at central and testicular levels [1, 4–6]. On the other hand, some nutrients such as PUFAs, minerals, and vitamins seem to improve the reproductive functions through several potential mechanisms of action [6–9]. For example, it is now known that the diets rich in long-chain PUFAs (≥20 C, LC-PUFAs) improve semen quality by modifying the fatty acid profile of plasma, seminal fluid, and sperm [3, 10–14].

Moreover, other nongenetic factors that can induce hypofertility and infertility include infections and inflammation of the genital tract [2, 15]. According to the World Health Organization [16], they are among the main causes for male infertility. In livestock farms such as rabbitries, clinical and subclinical infections of genital apparatus are not only a welfare issue but also a source of considerable economic losses due to reduced productivity, culling rate, purchase of drugs for treatments, and veterinarian consultations [2, 6, 17]. The genital infections in rabbit, but also in humans and other animal species, are commonly caused by Gram-negative bacteria such as *Pasteurella multocida*, *Escherichia coli*, *Pseudomonas* spp., and *Salmonella* spp. [15, 18–20]. The understanding of the body's defence mechanisms against bacteria could help in developing new strategies to treat infections and thus reduce infertility problems by using alternative therapeutic agents to antibiotics [20–22].

In mammals, invading pathogens are detected by innate immune system by pattern recognition receptors, among which Toll-like receptors (TLRs) are the most characterized. In particular, the TLR4 recognizes the lipopolysaccharides (LPS) of Gram-negative bacteria [21, 23, 24]. The activation of TLR4 after LPS binding stimulates an intracellular signalling cascade resulting in the activation of NF-*κ*B transcription factor that promotes the expression of immune response genes including those encoding proinflammatory cytokines and other mediators of inflammation [25–28]. Although the activation of TLR4 has a critical role in controlling the spread of invading pathogens in the genital tract, their overactivation may be dangerous for the host [17, 24, 29]. Indeed, TLR4 hyperactivation is involved in the development of several inflammatory pathologies and chronic diseases of the male genital tract. TLR4 has been localized in the human epididymis and prostate, where it can activate the inflammatory response and ultimately leads to temporary or persistent infertility [20, 29, 30]. However, its role in the switch from physiological to pathological condition of the genital tract, especially in the male, requires further studies [20, 24]. Moreover, relatively little is known about its expression and distribution in genital apparatus of the rabbit, although it is not only a livestock species but also a pet and an excellent animal model to study immunological aspects of the reproductive disorders [2, 21, 31–34]. Indeed, the rabbit TLR4 is more similar to the human one compared to mouse and rat [35].

Among the strategies to prevent and manage the infections of the genital tract, the use of some natural components in the diet, called functional foods or nutraceuticals, provides an attractive alternative to pharmacologic interventions. Indeed, experimental and epidemiological studies have shown the LC-PUFAs, in addition to their direct effects on sperm fatty acid profiles, have also anti-inflammatory properties [14, 36, 37]. In most of these studies, fish oil was used as source of eicosapentaenoic acid (EPA, 20:5n-3) and docosahexaenoic acid (DHA, 22:6n-3) [6, 14, 36, 37]. Nevertheless, LC-PUFAs can be also obtained indirectly from other sources like the linseed. In fact, linseed contains *α*-linolenic acid (ALA, 18:3n-3) which can be endogenously converted into EPA and DHA [14, 33, 36]. Mechanisms underlying the anti-inflammatory actions of PUFAs not only include the inhibition of the production of eicosanoids, like prostaglandins and leukotrienes, but also of proinflammatory cytokines such as TNF-*α*, IL-1*β*, IL-6, and IL-8 [37–40]. However, to our knowledge, no studies have investigated if n-3 PUFAs affect the inflammatory response of the male genital tract by modulating the TLR4 signalling pathway.

The first aim of this study was to elucidate the expression profiles of TLR4 and the proinflammatory cytokines TNF-*α* and IL-1*β* in the gonadal axis of male rabbit. Then, it investigated whether diet rich in different sources of n-3 PUFAs, extruded linseed and fish oil, affected their basal expression levels and fatty acid profile in seminal plasma.

## 2. Materials and Methods

### 2.1. Animals

Fifteen healthy New White Zealand bucks trained for semen collection, of the same body weight (4.5 ± 0.50 kg) and age (6 months old), were kept individually in flat deck cages and housed under controlled conditions: constant temperature of 21 ± 1°C, relative humidity of 55 ± 10%, and a continuous photoperiod of 16L:8D. Fresh water was always available *ad libitum*.

### 2.2. Experimental Design

Rabbits were randomly assigned to one of three groups (5 animals/group): C group was fed with commercial standard diet (lacking linseed or fish oil), L group with diet rich in extruded linseed (10%), and FO group with diet rich in fish oil (3.5% NORDIC NATURALS omega-3®) [14, 33]. Vitamin E (200 mg/kg feed, synthetic alpha-tocopheryl acetate) was included in all diets. Tables 1 and 2 show formulation and proximate analysis of the diets. The rabbits received each 130 g/d of feed for 110 days and then they were slaughtered according to the standard procedure practised in commercial abattoirs. Before slaughter, semen samples were collected following procedures previously reported [11] and immediately transferred to the laboratory. This study was conducted in accordance with the Guiding Principles in the Use of Animals and approved by the Animal Ethics Monitoring Committee of the University of Siena (CEL AOUS); Authorisation no. 265/2018-PR, ISOPRO 7DF19.23.

### 2.3. Fatty Acid Profile of Sperm

The total lipid extraction from the feed and semen was performed according to the method of Folch et al. [42], and the esterification was carried out following the procedure of Christie [43]. The transmethylation procedure was conducted using eicosenoic acid methyl esters (Sigma Chemical Co.) as internal standard. The recovery rates of the internal standard were 95 ± 1% and 89 ± 4% for feed and semen, respectively.

The fatty acid composition was determined using a Varian gas chromatograph (CP-3800) equipped with a flame ionisation detector and a capillary column of 100 m length × 0.25 mm × 0.2 *μ*m film (Supelco, Bellefonte, PA, USA). Helium was used as the carrier gas with a flow of 2 ml/min. The split ratio was 1 : 80.

Individual FAME was identified by comparing the relative retention times of peaks in the sample with those of standard mixture (FAME Mix Supelco; 4 : 0 to 24 : 0) plus cis-9, cis-12 C18:2; cis-9, cis-12, cis-15 C18:3; cis-9, cis-12, cis-15 C18:3 (all from Sigma-Aldrich).

### 2.4. Tissue Collection

The genital tract, hypothalamus, and pituitary were collected at the slaughterhouse. The male reproductive organs were immediately excised and subdivided into the testicles, epididymis, seminal vesicles, and prostate. Moreover, the brain and pituitary glands were promptly removed from each animal. The pituitary glands were bisected through the medial sagittal plane into 2 symmetrical parts. The brains were sectioned to isolate the diencephalic portions. Samples of organs were collected for histological and polymerase chain reaction (PCR) evaluation. The TLR4 and proinflammatory cytokine mRNA levels in the various organs of the reproductive tract and gonadal axis were evaluated using real-time PCR.

### 2.5. Histological Assay of Tissues

Samples from each rabbit of the testicle, epididymis, seminal vesicles, prostate, pituitary gland, and hypothalamus were collected. Tissues were fixed in 10% neutral buffered formalin, paraffin-embedded (FFPE), cut into 5 *μ*m sections, which were routinely stained with hematoxylin and eosin and examined by light microscopy.

### 2.6. RNA Extraction

Total RNA was extracted from formalin-fixed paraffin-embedded samples using the RNeasy FFPE Kit (Qiagen, Hilden, Germany) according to the manufacturer's protocol. Finally, the RNA concentration and quality were determined using a spectrophotometer (BioPhotometer, Eppendorf, Hamburg, Germany) at 260/280 nm wavelength. The samples were stored at -20°C.

### 2.7. Reverse Transcription and Real-Time PCR

Total RNA (1 *μ*g) from each sample was reverse-transcribed using the High-Capacity cDNA Reverse Transcription Kit (Applied Biosystems, Foster City, CA, USA), according to the manufacturer's instructions. The cDNA obtained from each sample was used as a template for Sybr Green dye-based (SYBR Green PCR Master Mix, Applied Biosystems, Foster City, CA, USA) Real-Time PCR in an optimized 20 *μ*l reaction volume in MicroAmp optical 96-well plates, as previously described [44]. Rabbit specific primers were purchased from Bio-Rad (Hercules, CA, USA). Their details are listed in [Supplementary-material supplementary-material-1].

A duplicate no-template control (NTC) was also included in each plate. Real-time quantitative PCR was carried out in the QuantStudio 3 Real-Time PCR Systems (Applied Biosystems, Foster City, CA, USA) at the following thermal cycle conditions: 2 minutes at 50°C, 10 minutes at 95°C followed by 50 cycles of 15 seconds at 95°C, and 1 minute at 60°C. Quantitation was determined after the application of an algorithm to the data analyzed by the software of the QuantStudio 3 Real-Time PCR Systems (Applied Biosystems, Foster City, CA, USA).

The expression of target genes (IL-1*β*, TNF-*α*, TLR4) was normalized using the calculated actin beta (housekeeping gene) cDNA expression (mean) of the same sample and run. The relative quantification of each gene was calculated using the formula 2^-*Δ*Ct^ where *Δ*Ct = Ct (target gene)–Ct (housekeeping gene), where Ct (threshold cycle) values were the mean of two test replicates. The obtained values were multiplied by 10,000 in order to obtain the test arbitrary units.

### 2.8. Statistical Analysis

Diagnostic graphs and the Shapiro-Wilk test were used to verify the assumptions of the parametric tests and identify outliers. Data on sperm fatty acid composition were analyzed by the one-way analysis of variance (ANOVA), followed by multiple comparisons with Sidak adjustment. Results were presented as means and pooled standard errors (SE). When the assumptions were not met (gene expression, [Supplementary-material supplementary-material-1]), values were log-transformed and a nonparametrical approach was chosen. Group differences were analyzed by using the Friedman tests treating the tissues as a within-subjects factor. Instead, differences between tissues were evaluated by the Kruskal-Wallis test using Dunn's test to carried out multiple comparisons. Data were expressed as a median value with interquartile range.

Statistical analyses were performed using SPSS 25.0 software (IBM, SPSS Inc., Chicago, IL, USA) and GraphPad Prism version 6.0 (San Diego, CA, USA). Statistical significance was set at *P* ≤ 0.05.

## 3. Results

### 3.1. Histological Tissues

**Figure 1:**
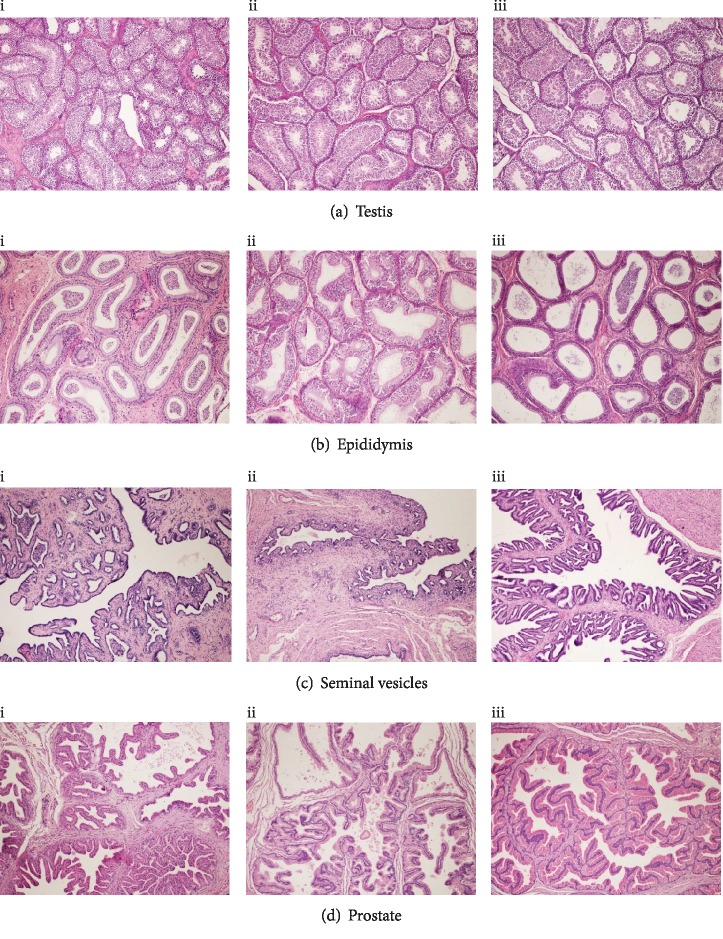
Seminiferous tubules of the testicles (a), tubules of the epididymis lined by ciliated tall columnar epithelium variably containing spermatozoa (b), glandular architecture of the seminal vesicles (c), and the prostate (d) from rabbit fed with control diet (C group, i), diet rich in linseed (L group, ii), or fish oil (FO group, iii). HE, 100x.

**Figure 2:**
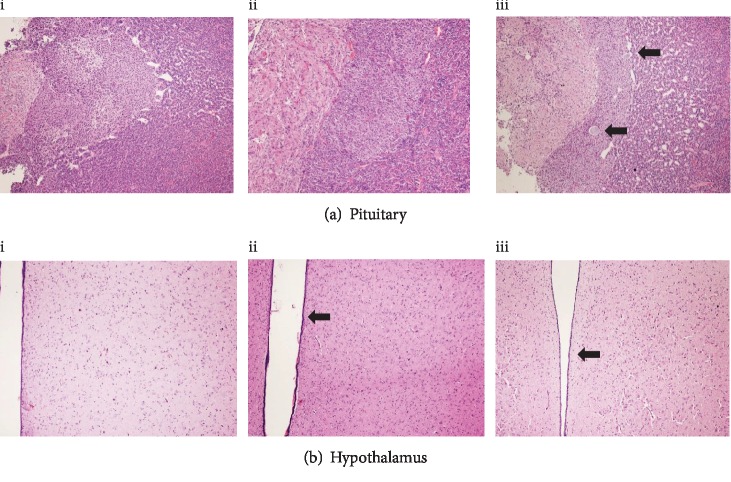
(a) Pituitary gland. Note the presence of multiple small cysts in the *pars intermedia* (arrows). (b) Hypothalamic areas where some nuclei are shown. The third ventricle is identified by the arrow. Rabbit fed with control diet (C group, i), diet rich in linseed (L group, ii), or fish oil (FO group, iii). HE, 100x.

Histological evaluation of sampled organs did not show differences between the 3 groups. Individual cell degeneration in glandular tissues or cystic degeneration of *pars intermedia* of the pituitary glands was noted randomly distributed in the groups (Figures 1 and 2).

### 3.2. TLR4 and Cytokine Expression Profile in the Gonadal Axis and Effect of Diet Enrichment with n-3 PUFA

**Figure 3:**
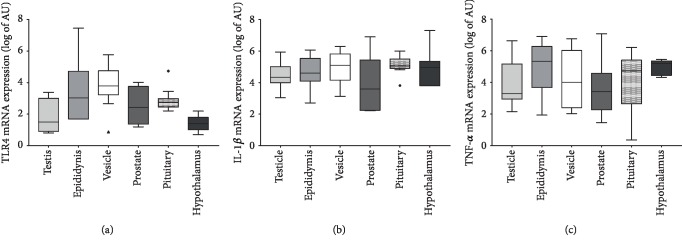
mRNA expression of TLR4 (a), IL-1*β* (b), and TNF-*α* (c) according to the tissues (regardless of the group). Values are log-transformed arbitrary units (AU). The symbols outside the box plot are outliers.

Differential expression of TLR4 along the gonadal axis was found in male rabbit (*P* < 0.05), although the prostate and testis were excluded from statistical analysis as they were detectable in an insufficient number of samples. In particular, TLR4 mRNA expression was lower in the hypothalamus than epididymis (*P* < 0.01), vesicle (*P* < 0.01), and pituitary gland (*P* < 0.05; Figure 3). Conversely, dietary treatment did not affect the expression of TLR4 (*P* > 0.1; Table 3).

There were no differences in TNF-*α* and IL-1*β* mRNA expression values either between tissues (*P* > 0.1; Figure 3) or groups (*P* > 0.1; Table 3).

### 3.3. Sperm Fatty Acid Composition

The diets altered fatty acid composition of the sperm (Table 4). Compared with the control group, rabbits fed with L and FO had lower n-6 (*P* < 0.05), LC-PUFA n-6 (*P* < 0.05), and n-6/n-3 ratio (*P* < 0.05). Conversely, the content of n-3 (*P* < 0.001) and LC-PUFA n-3 (*P* < 0.01) increased in the following order: the control < LI < FO group, mainly due to the DHA (22:6n-3) amount.

## 4. Discussion

There is a considerable body of evidence to support the fact that local and systemic infective and inflammatory disease may cause transient or permanent infertility in both humans and animals [2, 16, 20, 24].

Infections and inflammation can affect different sites of the male reproductive system, although the pathological modifications in sperm function are most probably due to direct effects of inflammatory mediators in the testis and accessory glands which induce damages, in particular, at seminiferous epithelium and vascular structures [20, 22, 45]. Moreover, there is evidence that bacterial LPS, proinflammatory cytokines, ROS, and nitric oxide may also determine infertility perturbing the secretion of hormones related to reproduction, acting at different levels of the gonadal axis [46–48]. The mechanisms of protection against the microbial agents are essential to maintain the male reproductive physiology. TLRs are a part of innate immune system and represent the link between innate and adaptive immunities. For this reason, they play a critical role in the activation of immune responses against infections [35]. TLRs have been characterized in different tissues and organs in human and different animal species [21, 24, 28]. However, studies about their expression and distribution in genital apparatus of the male are sparse and, as far as we know, this is the first study that tries to point out the map and the distribution of TLR4 and some proinflammatory cytokines in genital tract and gonadal axis on rabbit buck.

Our findings provide evidence that the mRNA encoding TLR4 is present in the testis, epididymis, seminal vesicles, prostate, pituitary, and hypothalamus of male rabbit although at different levels. In fact, TLR4 mRNA was not found in the testis and prostate of all samples analyzed and showed weak expression in the hypothalamus. Conversely, it was predominantly expressed in the epididymis and seminal vesicles.

Differences in TLR4 expression throughout the gonadal axis and genital tract suggest a tissue-specific immune surveillance analogous to that hypothesized for humans. In fact in humans, it is low in the prostate and testis, as well as in the brain [21, 35, 49]. Conversely, it could play an important role in defending against *Chlamydia* and *E. coli* infections in the epididymis [20, 22, 30] while seminal vesicles have been poorly studied.

Some authors also pointed out that TLR4 expression in the genital tract is different between the species, but these differences, as well as those between tissues, could simply be due to the lack of tools for analysis or lack of information [21]. Indeed, as mentioned above, TLR4 have been identified and localized in several tissues of male reproductive tract of rodents [22, 24, 35, 50] but information about nonhuman primates, swine, and dog is scarce [35]. Really, expression of TLR4 mRNA in the human epididymis has just been documented by Saeidi et al. [30] while it has only recently been located in the epididymal duct, vas deferens, and sperm of cats by immunohistochemistry and western blotting [51].

In rabbit, high levels of TLR4 expression have been found in the lung and bone marrow [28, 35]. Even if moderately, it has been also detected in the kidney, spleen, heart, liver, and thymus [35]. Collodel et al. [8] showed that the TLR-4 was expressed in the testis and epididymis, but, to our knowledge, this study is the first that investigates the expression in the whole male reproductive hypothalamic-gonadal axis of rabbit. It is interesting to note that rabbit is a better animal model for the study of TLR4 compared to rodents, sharing a 72% amino acid similarity with humans [35].

Although a differential spatial distribution was observed, the ubiquitous expression of TLR4 supports the idea that the male reproductive tract is an aseptic site in an active surveillance state and that TLR4 plays a primary role in the immune response to an infection. Rabbit genital infections are mainly caused by Gram-negative bacteria such as *Pasteurella multocida*, *Escherichia coli*, *Pseudomonas* spp., and *Salmonella* spp. as a result of incorrect artificial insemination practices and poor environmental hygiene [15, 19]. TLR4 in complex with some accessory molecules such as CD14 and MD-2 can bind LPS, the major component of the outer membrane of Gram-negative bacteria [21, 23, 27]. Binding of LPS to TLR4 triggers the intracellular transduction events leading to the activation of different transcription factors. In particular, two different pathways can be activated: MyD88-dependent and MyD88-independent. The first pathway leads to the activation of NF-*κ*B that in turn promotes the expression of proinflammatory cytokines such as IL-1*β*, TNF-*α*, and IL-6. MyD88-independent pathway, through the involvement of TRAM and TRIF adapter molecules, leads to the activation of IRF3 and the following expression of IFNs [27, 28, 35]. The activation of TLR4 also stimulates the production of other mediators of inflammation including ROS, nitric oxide, and antimicrobial factors such as defensins and heat shock proteins [25, 26, 47].

Our study demonstrated the ubiquitous expression of the proinflammatory cytokines IL-1*β* and TNF-*α* through the reproductive hypothalamic-gonadal axis of male rabbit without, moreover, highlighting differences between tissues.

Although the activation of TLR4, and the expression of IL-1*β* and TNF-*α*, provides an essential host immune defence mechanism, they are also involved in different aspects of infertility. Local production of the cytokines, in particular IL-1*β* and TNF-*α*, affects the blood supply of the testis, may inhibit Leydig cell steroidogenesis, and in turn affects spermatogenesis [24, 45, 46]. Thus, inflammatory mediators have a direct effect on the seminiferous epithelium and testicular vasculature, thus compromising the fertility. Moreover, they may contribute to the detrimental effects of epididymitis, prostatitis, and, probably, of prostate cancer upon male fertility [20, 24, 29]. Finally, ROS produced by immune and nonimmune cells during the inflammatory reaction can determine an oxidative damage of spermatozoa, lipid, and protein peroxidation, as well as DNA fragmentation, resulting in the loss of sperm functions [6, 13, 48]. Furthermore, there is a considerable body of evidence suggesting that local and systemic infections and inflammation may also act on the gonadal axis [52]. This alteration is mainly due to a reduction in the secretion of important hormones related to reproduction. It is evident that bacterial lipopolysaccharides, proinflammatory cytokines, and other inflammatory mediators such as ROS and nitric oxide can impair the hormonal secretion at different levels of the brain-pituitary-Leydig cell axis [45, 46].

In the last decades, the interest in the nutrition as a modifiable factor in immune function has growing. In particular, several indirect evidences suggest that n-3 PUFAs can modulate the immune response. For example, the ratio between n-6 to n-3 PUFAs in the diet is related to an increased risk of inflammatory chronic disease incidence and benefits of n-3 PUFA have been demonstrated in rheumatoid arthritis, inflammatory bowel disease, and asthma [36, 37]. In addition to their role as precursors of eicosanoids, n-3 PUFA have been demonstrated to modulate the inflammatory response attenuating the activation of the TLR4 signalling and inhibiting the expression of IL-1*β*, TNF-*α*, IL-6, NF-*κ*B, and reactive oxygen and nitrogen species [37–40].

In light of these considerations, this study for the first time examined the effects of n-3 PUFA-enriched diets in the TLR4 signalling pathway of the male genital tract of the rabbit. The animals did not show any clinical signs throughout the period of the treatment and, both histological evaluation and quantification of gene expression, did not reveal differences on gonadal axis and genital tract due to PUFA enrichment.

The anti-inflammatory effects of LC-PUFA on the reproductive system have previously been evaluated in females with conflicting results depending on the tissue, species, and experimental design. DHA treatment attenuates inflammatory responses modulating the TLR4 signalling pathway in mammary tissues of saws [53], but incubation with LC-PUFA did not modify proinflammatory cytokine response in human placental cells [54]. LC-PUFA supplementation during pregnancy reduced inflammation in placental tissue of mice after bacterial induction of intrauterine infection [55] and in human adipose tissue and trophoblast cells [56]. TLR4 plays a key role in determining these anti-inflammatory effects [55, 56]. Our finding did not show an effect of diets enriched in LC-PUFA on the basal expression of TLR4 and cytokines through the hypothalamic-gonadal axis of male rabbit, but further studies could assess their levels after LPS stimulation.

Conversely, as expected, sperm fatty acid composition was altered by dietary treatment. Increased n-3 and LC-PUFA n-3 were found in rabbits supplemented with linseed and fish oil while their n-6/n-3 ratio was considerably reduced. These findings confirm that sperm fatty acid profile of the rabbit is very sensitive to the diet rich in n-3 PUFAs [11]. Moreover, they suggest that n-3 PUFAs have beneficial effects on male fertility thanks to their impacts on the sperm structure.

Indeed, the type of PUFAs has strong effects on maintaining the integrity and fluidity of the sperm membrane, on membrane protein and ion channel function, on regulation of nuclear receptors, and therefore on the morphology, motility, and acrosome reaction of the sperm [3, 10, 11]. Furthermore, it has been suggested that the changes of PUFA composition on sperm by dietary fatty acids may also induce changes in metabolism and enzyme potential of germinal cells that influence the fertility [5].

However, in the present study, no lineal relationship exists between n-3 PUFA ingestion and their profile in the sperm: it seems that n-3 PUFAs from fish oil were more effective with respect to those provided by the linseed. This is justifiable by the fatty acid composition of rabbit sperm: membrane phospholipids were constituted by 43% of LC-PUFAs, and then they incorporate more easily EPA, DHA, and especially the docosapentaenoic acids from n-6 series (22:5n-6) with respect to their precursors LA and ALA (n-6 and n-3, respectively) [14].

Finally, PUFAs may influence the secretion of LH, FSH, and testosterone, although not all studies agree [7, 10, 12]. Improvement of membrane fluidity, motility, and fertility linked to dietary enrichment in PUFA and then to changes in sperm composition have been widely documented in humans and in several animal species [3, 7, 10–13].

However, the manipulation of the diet not always coincides with an improvement of sperm parameters and fertility [6, 13]. Different experimental procedures in terms of time and dose of administration might explain these responses. It is very important to note that an excessive fluidity can damage the plasma membrane leading to a decrease in the cell signal transduction with detrimental effects on the sperm [6, 12]. Moreover, PUFA are subjected to the attack of ROS that induces the lipid peroxidation of plasma membrane eventually resulting in the impairment of membrane fluidity, permeability, function of ion channels and receptors, and then to sperm functions [6, 11, 13]. In our study, an overnutritional amount of vitamin E was added to avert this danger [11, 13, 14]. Moreover, flaxseed also contains lignan, which is a kind of phytoestrogens that can act as an antioxidant important to increase the stability of PUFA in sperm and seminal plasma and, at the same time, reduce the production and the effects of ROS [11, 33]. Then, n-3 PUFAs on one hand can counteract oxidative effects of ROS but at same time, when excessive, can themselves be sources of ROS. In other words then, the dietary enrichment in n-3-PUFAs may improve semen quality given that its quantity is controlled so to avoid lipid peroxidation.

Indeed, two main limitations should be pointed out in the current study which could be subject to future research: (i) to investigate the effect of diets with different levels of PUFAs and, consequently, also different fatty acid compositions of sperm, and (ii) to investigate whether the diets enriched in PUFAs influence the expression of TLR4 and cytokines through the hypothalamic-gonadal axis of male rabbit after a proinflammatory challenge.

## 5. Conclusions

In this study, we pointed out, for the first time, the map of distribution of TLR4 and proinflammatory cytokines such as IL-1*β* and TNF-*α* in the reproductive organs and gonadal axis of the male rabbit. This knowledge may be important to understand the primary immune response sites to an invading pathogen agent in the reproductive tract and the mechanisms by which the consequent inflammation can induce infertility.

Our results showed that the diets rich in n-3 PUFAs do not affect the basal expression of TLR4 and cytokines in the genital tract. Future directions exploring their effects on TLR4 signalling pathway after LPS stimulation to eventually implement specific nutritional protocols, particularly in the setting of inflammatory diseases, are needed.

However, the reduced n-6/n-3 ratio on seminal plasma of rabbits supplemented with linseed or fish oil indicates that n-3 PUFAs could improve reproductive functions by modifying the sperm lipid composition.

Finally, our data suggest that the rabbit could be a useful animal model to understand the relationship between inflammation and infertility as well as to understand the mechanisms through which PUFAs may influence the male fertility.

## Figures and Tables

**Table 1:** Formulation and chemical composition of control (C) and enriched diets with extruded linseed (L) or fish oil (FO).

	Unit	Diet		
		C	L	FO
*Ingredients*				
Dehydrated alfalfa meal	g/kg	300	380	380
Soybean meal 44%	g/kg	150	100	150
Barley meal	g/kg	410	310	335
Wheat bran	g/kg	52	52	52
Soybean oil	g/kg	30	—	—
Extruded flaxseed	g/kg	—	100	—
Fish oil^∗^	g/kg	—	—	35
Beet molasses	g/kg	20	10	10
Calcium carbonate	g/kg	7	7	7
Calcium diphosphate	g/kg	13.5	13.5	13.5
Salt	g/kg	7	7	7
DL-methionine	g/kg	0.5	0.5	0.5
Vitamin-mineral premix^†^	g/kg	10	10	10
*Analytical data*				
Crude protein	g/kg	175	174	175
Ether extract	g/kg	480	472	425
Crude fiber	g/kg	124	137	130
Ash	g/kg	89	84	90
*Digestible energy* ^§^	MJ/kg f.m.	10.6	10.7	10.9

^∗^NORDIC NATURALS omega-3®: purified deep sea fish oil (from anchovies and sardines) containing EPA 330 mg/100 g, DHA 220 mg/100 g, other n-3 LC-PUFA 140 mg/100 g+*α*-tocopherol for preservation. ^†^Per kg diet: vitamin A 11.000 IU; vitamin D_3_ 2000 IU; vitamin B_1_ 2.5 mg; vitamin B_2_ 4 mg; vitamin B_6_ 1.25 mg; vitamin B_12_ 0.01 mg; alpha-tocopheryl acetate 200 mg; biotin 0.06 mg; vitamin K 2.5 mg; niacin 15 mg; folic acid 0.30 mg; D-pantothenic acid 10 mg; choline 600 mg; Mn 60 mg; Fe 50 mg; Zn 15 mg; I 0.5 mg; Co 0.5 mg; ^§^Maertens et al. 1988 [41].

**Table 2:** Main fatty acid composition (expressed as percentage of total fatty acids) of diets.

Fatty acids	Diets			Pooled SE
	C	L	FO	
SFA	19.80_a_	15.40_a_	38.10_b_	1.82
MUFA	17.40	15.80	14.50	0.87
PUFA	62.80_a_	68.80_a_	47.40_b_	5.12
C18:2n-6, LA	50.45_b_	22.30_a_	20.50_a_	2.11
C18:3n-3, ALA	11.15_a_	45.80_b_	18.50_a_	1.42
C20:5n-3, EPA	—	—	3.50	0.21
C22:6n-3, DHA	—	—	4.20	0.28
LC-PUFAn-3	—	—	10.50	1.00
n-6	51.45_b_	22.80_a_	21.00_a_	2.35
n-3	11.35_a_	46.00_c_	26.40_b_	1.55
n-6/n-3	4.53_b_	0.50_a_	0.80_a_	0.01

SFA: saturated fatty acids; MUFA: monounsaturated fatty acids; PUFA: polyunsaturated fatty acids; LA: linoleic acid; ALA: *α*-linolenic acid; LC-PUFA: long-chain PUFA; EPA: eicosapentaenoic acid; DHA: docosahexaenoic acid. Values followed by the same subscript letter in each row do not differ significantly (*P* ≤ 0.05).

**Table 3:** Marginal medians and interquartile range of the normalized expression values of TLR4, IL-1*β*, and TNF-*α* according to group (regardless of the tissue). C: control diet; L: diet rich in extruded linseed; FO: diet rich in fish oil.

Gene target	Group			*P* value^∗^
	C	L	FO	
TLR4	2.67 (1.55-3.81)	3.03 (1.70-4.73)	2.59 (1.50-2.96)	0.653
IL-1*β*	4.26 (3.59-5.13)	4.93 (4.34-5.16)	5.19 (3.95-5.68)	0.273
TNF-*α*	3.35 (2.85-4.65)	4.49 (3.34-5.34)	5.23 (3.29-6.06)	0.653

^∗^From Friedman test.

**Table 4:** Polyunsaturated fatty acid composition (expressed as percentage of total fatty acids) of sperm. C: control diet; L: diet rich in extruded linseed; FO: diet rich in fish oil.

	Experimental diets			Pooled SE	*P* value
Fatty acids	C	L	FO		
C18:2n-6, LA	7.2_a_	3.8_b_	5.7_b_	2.09	0.033
C20:4n-6, ARA	1.5_a_	0.7_b_	0.7_b_	0.09	0.026
C22:5n-6	26.2_a_	17.9_b_	12.7_b_	3.92	0.021
n-6	34.9_a_	22.4_b_	19.1_b_	3.85	0.017
C18:3n-3, LA	0.2_a_	1.3_c_	0.5_b_	0.11	0.020
C20:5n-3, EPA	0.2_a_	0.5_a_	5.3_b_	0.10	0.011
C22:6n-3, DHA	0.3_a_	2.4_b_	7.6_c_	1.25	<0.001
n-3	0.7_c_	4.2_b_	13.4_a_	0.21	<0.001
n6/n-3	49.9_a_	5.4_b_	2.1_b_	15.8	0.021
LC-PUFA n-3	0.5_c_	2.9_b_	12.9_a_	0.26	0.005
LC-PUFA n-6	27.7_a_	18.4_b_	14.4_c_	1.19	0.032

SE: standard error. *P* value: significance value of *F* ratio (ANOVA test which compares the three group means). Values in the same row not sharing the same subscript are significantly different (*P* ≤ 0.05; multiple comparisons with Sidak correction).

## Data Availability

The data used to support the findings of this study are included within the article and the supplementary information file. Additional information or data sets are available from the corresponding author upon request.

## Supplementary Materials

Supplementary 1 Table S1: primer list.

Supplementary 2 Table S2: mRNA expression of TLR4, IL-1*β*, and TNF-*α* (arbitrary units, untransformed data).

## References

[1] Vander Borght M., Wyns C. (2018). Fertility and infertility: definition and epidemiology. *Clinical Biochemistry*.

[2] Brecchia G., Menchetti L., Cardinali R. (2014). Effects of a bacterial lipopolysaccharide on the reproductive functions of rabbit does. *Animal Reproduction Science*.

[3] Alonge S., Melandri M., Leoci R., Lacalandra G. M., Caira M., Aiudi G. G. (2019). The effect of dietary supplementation of vitamin E, selenium, zinc, folic acid, and N-3 polyunsaturated fatty acids on sperm motility and membrane properties in dogs. *Animals*.

[4] Brecchia G., Bonanno A., Galeati G. (2006). Hormonal and metabolic adaptation to fasting: effects on the hypothalamic-pituitary-ovarian axis and reproductive performance of rabbit does. *Domestic Animal Endocrinology*.

[5] Chavarro J. E., Mínguez-Alarcón L., Mendiola J., Cutillas-Tolín A., López-Espín J. J., Torres-Cantero A. M. (2014). Trans fatty acid intake is inversely related to total sperm count in young healthy men. *Human Reproduction*.

[6] Wathes D. C., Abayasekara D. R. E., Aitken R. J. (2007). Polyunsaturated fatty acids in male and female reproduction. *Biology of Reproduction*.

[7] Feng Y., Ding Y., Liu J. (2015). Effects of dietary omega-3/omega-6 fatty acid ratios on reproduction in the young breeder rooster. *BMC Veterinary Research*.

[8] Collodel G., Moretti E., del Vecchio M. T. (2014). Effect of chocolate and propolfenol on rabbit spermatogenesis and sperm quality following bacterial lipopolysaccharide treatment. *Systems Biology in Reproductive Medicine*.

[9] Menchetti L., Vecchione L., Filipescu I. (2019). Effects of Goji berries supplementation on the productive performance of rabbit. *Livestock Science*.

[10] Esmaeili V., Shahverdi A. H., Moghadasian M. H., Alizadeh A. R. (2015). Dietary fatty acids affect semen quality: a review. *Andrology*.

[11] Mourvaki E., Cardinali R., Dal Bosco A., Corazzi L., Castellini C. (2010). Effects of flaxseed dietary supplementation on sperm quality and on lipid composition of sperm subfractions and prostatic granules in rabbit. *Theriogenology*.

[12] Safari Asl R., Shariatmadari F., Sharafi M., Karimi Torshizi M. A., Shahverdi A. (2018). Improvements in semen quality, sperm fatty acids, and reproductive performance in aged Ross breeder roosters fed a diet supplemented with a moderate ratio of n-3: N-6 fatty acids. *Poultry Science*.

[13] Liu Q., Zhou Y. F., Duan R. J., Wei H. K., Jiang S. W., Peng J. (2015). Effects of dietary n-6:n-3 fatty acid ratio and vitamin E on semen quality, fatty acid composition and antioxidant status in boars. *Animal Reproduction Science*.

[14] Rodríguez M., Rebollar P. G., Mattioli S., Castellini C. (2019). n-3 PUFA sources (precursor/products): a review of current knowledge on rabbit. *Animals*.

[15] Boiti C., Canali C., Brecchia G., Zanon F., Facchin E. (1999). Effects of induced endometritis on the life-span of corpora lutea in pseudopregnant rabbits and incidence of spontaneous uterine infections related to fertility of breeding does. *Theriogenology*.

[16] World Health Organization (2000). *WHO manual for the standardized investigation and diagnosis of the infertile couple*.

[17] Menchetti L., Barbato O., Filipescu I. E. (2018). Effects of local lipopolysaccharide administration on the expression of Toll-like receptor 4 and pro-inflammatory cytokines in uterus and oviduct of rabbit does. *Theriogenology*.

[18] Pergola S., Franciosini M. P., Comitini F. (2017). Genetic diversity and antimicrobial resistance profiles of Campylobacter coli and Campylobacter jejuni isolated from broiler chicken in farms and at time of slaughter in Central Italy. *Journal of Applied Microbiology*.

[19] Brecchia G., Cardinali R., Mourvaki E. (2010). Short- and long-term effects of lipopolysaccharide-induced inflammation on rabbit sperm quality. *Animal Reproduction Science*.

[20] Silva E. J. R., Ribeiro C. M., Mirim A. F. M. (2018). Lipopolysaccharide and lipotheicoic acid differentially modulate epididymal cytokine and chemokine profiles and sperm parameters in experimental acute epididymitis. *Scientific Reports*.

[21] Jungi T. W., Farhat K., Burgener I. A., Werling D. (2011). Toll-like receptors in domestic animals. *Cell and Tissue Research*.

[22] Mackern-Oberti J. P., Motrich R. D., Breser M. L., Sánchez L. R., Cuffini C., Rivero V. E. (2013). *Chlamydia trachomatis* infection of the male genital tract: An update. *Journal of Reproductive Immunology*.

[23] Filipescu I. E., Leonardi L., Menchetti L. (2018). Preventive effects of bovine colostrum supplementation in TNBS-induced colitis in mice. *PLoS One*.

[24] Kannaki T. R., Shanmugam M., Verma P. C. (2011). Toll-like receptors and their role in animal reproduction. *Animal Reproduction Science*.

[25] Boiti C., Guelfi G., Zerani M., Zampini D., Brecchia G., Gobbetti A. (2004). Expression patterns of cytokines, p53 and nitric oxide synthase isoenzymes in corpora lutea of pseudopregnant rabbits during spontaneous luteolysis. *Reproduction*.

[26] Boiti C., Guelfi G., Zampini D., Brecchia G., Gobbetti A., Zerani M. (2003). Regulation of nitric oxide synthase isoforms and role of nitric oxide during prostaglandin F2alpha-induced luteolysis in rabbits. *Reproduction*.

[27] Kawai T., Akira S. (2010). The role of pattern-recognition receptors in innate immunity: update on toll-like receptors. *Nature Immunology*.

[28] Turin L., Riva F. (2008). Toll-like receptor family in domestic animal species. *Critical Reviews in Immunology*.

[29] Ou T., Lilly M., Jiang W. (2018). The pathologic role of toll-like receptor 4 in prostate cancer. *Frontiers in Immunology*.

[30] Saeidi S., Shapouri F., Amirchaghmaghi E. (2014). Sperm protection in the male reproductive tract by Toll-like receptors. *Andrologia*.

[31] Menchetti L., Brecchia G., Canali C. (2015). Food restriction during pregnancy in rabbits: effects on hormones and metabolites involved in energy homeostasis and metabolic programming. *Research in Veterinary Science*.

[32] Menchetti L., Brecchia G., Cardinali R., Polisca A., Boiti C. (2015). Feed restriction during pregnancy: effects on body condition and productive performance of primiparous does. *World Rabbit Science*.

[33] Menchetti L., Canali C., Castellini C., Boiti C., Brecchia G. (2018). The different effects of linseed and fish oil supplemented diets on insulin sensitivity of rabbit does during pregnancy. *Research in Veterinary Science*.

[34] Menchetti L., Brecchia G., Branciari R. (2020). The effect of Goji berries (*Lycium barbarum*) dietary supplementation on rabbit meat quality. *Meat Science*.

[35] Vaure C., Liu Y. (2014). A comparative review of toll-like receptor 4 expression and functionality in different animal species. *Frontiers in Immunology*.

[36] Zárate R., el Jaber-Vazdekis N., Tejera N., Pérez J. A., Rodríguez C. (2017). Significance of long chain polyunsaturated fatty acids in human health. *Clinical and Translational Medicine*.

[37] Calder P. C. (2015). Marine omega-3 fatty acids and inflammatory processes: effects, mechanisms and clinical relevance. *Biochimica et Biophysica Acta (BBA) - Molecular and Cell Biology of Lipids*.

[38] Robertson R., Guihéneuf F., Bahar B. (2015). The anti-inflammatory effect of algae-derived lipid extracts on lipopolysaccharide (LPS)-stimulated human THP-1 macrophages. *Marine Drugs*.

[39] Weldon S. M., Mullen A. C., Loscher C. E., Hurley L. A., Roche H. M. (2007). Docosahexaenoic acid induces an anti-inflammatory profile in lipopolysaccharide-stimulated human THP-1 macrophages more effectively than eicosapentaenoic acid. *The Journal of Nutritional Biochemistry*.

[40] Lee J. Y., Plakidas A., Lee W. H. (2003). Differential modulation of Toll-like receptors by fatty acids: preferential inhibition by n-3 polyunsaturated fatty acids. *Journal of Lipid Research*.

[41] Maertens L., Moermans R., De Groote G. (1988). Prediction of the apparent digestible energy content of commercial pelleted feeds for rabbits. *The Journal of Applied Rabbit Research*.

[42] Folch J., Lees M., Stanley G. S. (1957). A simple method for the isolation and purification of total lipides from animal tissues. *The Journal of Biological Chemistry*.

[43] Christie W. W. (1982). A simple procedure for rapid transmethylation of glycerolipids and cholesteryl esters. *Journal of Lipid Research*.

[44] Curone G., Filipe J., Cremonesi P. (2018). What we have lost: mastitis resistance in Holstein Friesians and in a local cattle breed. *Research in Veterinary Science*.

[45] O’Bryan M. K., Schlatt S., Phillips D. J., De Kretser D. M., Hedger M. P. (2000). Bacterial lipopolysaccharide-induced inflammation compromises testicular function at multiple levels in vivo. *Endocrinology*.

[46] Hong C. Y., Park J. H., Ahn R. S. (2004). Molecular mechanism of suppression of testicular steroidogenesis by proinflammatory cytokine tumor necrosis factor alpha. *Molecular and Cellular Biology*.

[47] O’Bryan M. K., Schlatt S., Gerdprasert O., Phillips D. J., de Kretser D. M., Hedger M. P. (2000). Inducible nitric oxide synthase in the rat testis: evidence for potential roles in both normal function and inflammation-mediated infertility. *Biology of Reproduction*.

[48] Agarwal A., Saleh R. A. (2002). Role of oxidants in male infertility: rationale, significance, and treatment. *The Urologic Clinics of North America*.

[49] Nishimura M., Naito S. (2005). Tissue-specific mRNA expression profiles of human Toll-like receptors and related genes. *Biological & Pharmaceutical Bulletin*.

[50] Biswas B., Narmadha G., Choudhary M., French F. S., Hall S. H., Yenugu S. (2009). Identification of toll-like receptors in the rat (rattus norvegicus): messenger RNA expression in the male reproductive tract under conditions of androgen variation. *American Journal of Reproductive Immunology*.

[51] Liman N., Alan E., Apaydın N. (2019). The expression and localization of Toll-like receptors 2, 4, 5 and 9 in the epididymis and vas deferens of a adult tom cats. *Theriogenology*.

[52] Reddy M. M., Mahipal S. V. K., Subhashini J. (2006). Bacterial lipopolysaccharide-induced oxidative stress in the impairment of steroidogenesis and spermatogenesis in rats. *Reproductive Toxicology*.

[53] Lin S., Zhang Y., Long Y. (2016). Mammary inflammatory gene expression was associated with reproductive stage and regulated by docosahexenoic acid: in vitro and in vivo studies. *Lipids in Health and Disease*.

[54] Yang X., Haghiac M., Glazebrook P., Minium J., Catalano P. M., Hauguel-de Mouzon S. (2015). Saturated fatty acids enhance TLR4 immune pathways in human trophoblasts. *Human Reproduction*.

[55] Garcia-So J., Zhang X., Yang X. (2019). Omega-3 fatty acids suppress Fusobacterium nucleatum–induced placental inflammation originating from maternal endothelial cells. *JCI Insight*.

[56] Haghiac M., Yang X., Presley L. (2015). Dietary omega-3 fatty acid supplementation reduces inflammation in obese pregnant women: a randomized double-blind controlled clinical trial. *PLoS One*.

