# Supplementary material for: Effects of Diets Enriched in Linseed and Fish Oil on the Expression Pattern of Toll-Like Receptors 4 and Proinflammatory Cytokines on Gonadal Axis and Reproductive Organs in Rabbit Buck
Source: Oxid Med Cell Longev. 2020 Jan 21;2020:4327470. doi: 10.1155/2020/4327470 (PMC6996712; doi:10.1155/2020/4327470)
Supplement: Supplementary 1 — Table S1: primer list. [file 4327470.f1.pdf]

Table S1. Primer list

PrimePCR™ SYBR® Green Assay	UniqueAssayID (Bio-Rad)
TNF, Rabbit	qOcuCED0015500
IL1B, Rabbit	qOcuCED0009632
TLR4, Rabbit	qOcuCID0002193
ACTB, Rabbit	qOcuCED0014181
