# Supplementary material for: Effects of Diets Enriched in Linseed and Fish Oil on the Expression Pattern of Toll-Like Receptors 4 and Proinflammatory Cytokines on Gonadal Axis and Reproductive Organs in Rabbit Buck
Source: Oxid Med Cell Longev. 2020 Jan 21;2020:4327470. doi: 10.1155/2020/4327470 (PMC6996712; doi:10.1155/2020/4327470)
Supplement: Supplementary 2 — Table S2: mRNA expression of TLR4, IL-1β, and TNF-α (arbitrary units, untransformed data). [file 4327470.f2.pdf]

Table S2. mRNA expression of TLR4, IL-1β, and TNF-α (Arbitrary Units, untransformed data) in hypothalamus, pituitary gland, testicle, epididymis, vesicle, and prostate of rabbits fed with standard diet (C), with a diet rich in extruded linseed (L), and with a diet rich in fish oil (FO). Values are medians and interquartile ranges.

Group	Gene target	TISSUE					
		**HYPOTHALAMUS**	**PITUITARY**	**TESTICLE**	**EPIDIDYMIS**	**VESICLE**	**PROSTATE**
**C**	**TLR4**	25.1 (5.0- 158.5)	562.3 (380.2- 660.7)	1230.7 (6.6- 2454.7)	12589.3 (50.1- 165958.7)	6760.8 (6166.0- 54954.1)	46.7 (15.8- 77.6)
	**IL-1β**	9983374.3 (14125.4- 19952623.2)	134896.3 (85113.8- 208929.6)	23442.3 (15135.6- 223872.1)	18197.0 (1000.0- 39810.7)	15676.9 (8242.3- 453734.5)	2028.2 (166.0- 3890.5)
	**TNF-α**	102883.0 (20809.0- 184957.0)	47819.1 (339.4- 199666.3)	897.0 (724.0- 936.0)	2622786.9 (2432.5- 6755332.8)	1263.0 (1259.0- 8471.0)	2127.5 (348.0- 3907.0)
**L**	**TLR4**	26.9 (24.5- 123.0)	1496.1 (168.0- 28260.8)	15.8 (15.8- 15.8)	1071.5 (50.1- 28183829.3)	56234.1 (6166.0- 575439.9)	10471.3 (10471.3- 10471.3)
	**IL-1β**	91201.1 (6456.5- 107151.9)	83198.4 (72924.2- 343836.7)	27118.5 (15938.8- 67344.3)	189483.4 (113860.2- 552693.2)	134144.0 (58227.3- 480143.5)	3981.1 (166.0- 87096.4)
	**TNF-α**	134061.9 (30715.0- 278754.0)	16431.9 (1121.8- 92549.6)	20997.1 (2442.1- 2194592.5)	111393.4 (4355.3- 754166.6)	383778.7 (122.7- 3268280.9)	2765.0 (28.0- 5502.0)
**FO**	**TLR4**	18.2 (5.9- 31.6)	562.3 (331.1- 660.7)	63.1 (63.1- 63.1)	1071.5 (50.1- 16595.9)	467.7 (7.6- 6166.0)	912.0 (912.0- 912.0)
	**IL-1β**	115164.3 (6456.5- 223872.1)	214214.8 (59329.2- 646732.5)	11201.1 (5004.5- 432313.8)	248206.4 (13246.2- 826763.8)	258979.9 (84342.8- 1179170.2)	389045.1 (213.8- 7943282.3)
	**TNF-α**	106113.0 (26.0- 212200.0)	258432.3 (73180.8- 1016314.8)	72445.0 (1879.6- 143583.0)	347043.0 (5361.2- 1875558.0)	1142657.6 (193638.2- 2730308.8)	6019491.4 (1768.0- 12037214.8)
